# The impact of a supportive supervision intervention on health workers in Niassa, Mozambique: a cluster-controlled trial

**DOI:** 10.1186/s12960-017-0213-4

**Published:** 2017-09-02

**Authors:** Tavares Madede, Mohsin Sidat, Eilish McAuliffe, Sergio Rogues Patricio, Ogenna Uduma, Marie Galligan, Susan Bradley, Isabel Cambe

**Affiliations:** 1 Department of Community Health, Faculty of Medicine, Eduardo Mondlane University, 702 Salvador Allende Ave., PO Box 257, Maputo, Mozambique; 2 School of Nursing, Midwifery and Health Systems, University College Dublin, Dublin, Ireland; 3 Centre for Global Health, Trinity College Dublin, Dublin, Ireland; 4 School of Medicine, University College Dublin, Dublin, Ireland; 5 School of Health Sciences, City University London, 1 Myddelton Street, London, EC1R 1UW United Kingdom; 6 National Health Institute, Maputo, Mozambique

**Keywords:** Supportive supervision, Job satisfaction, Retention, Work engagement, Burnout, Participation, Motivation

## Abstract

**Background:**

Regular supportive supervision is critical to retaining and motivating staff in resource-constrained settings. Previous studies have shown the particular contribution that supportive supervision can make to improving job satisfaction amongst over-stretched health workers in such settings.

**Methods:**

The Support, Train and Empower Managers (STEM) study designed and implemented a supportive supervision intervention and measured its’ impact on health workers using a controlled trial design with a three-arm pre- and post-study in Niassa Province in Mozambique. Post-intervention interviews with a small sample of health workers were also conducted.

**Results:**

The quantitative measurements of job satisfaction, emotional exhaustion and work engagement showed no statistically significant differences between end-line and baseline. The qualitative data collected from health workers post the intervention showed many positive impacts on health workers not captured by this quantitative survey.

**Conclusions:**

Health workers perceived an improvement in their performance and attributed this to the supportive supervision they had received from their supervisors following the intervention. Reports of increased motivation were also common. An unexpected, yet important consequence of the intervention, which participants directly attributed to the supervision intervention, was the increase in participation and voice amongst health workers in intervention facilities.

## Background

Ongoing support is needed for health workers in the frontline of service delivery to perform to their full potential and deliver quality patient care. In a discrete choice study of more than 2000 health workers across Tanzania, Malawi and Mozambique [1], good functioning human resource management systems emerged as a critical factor in job choice across all grades of health workers. Supervision and feedback on performance is an important component of Human Resource Management as it plays a key role in motivating staff. In a previous study, multi-level analysis of data from obstetric services in Malawi, Tanzania and Mozambique showed that negative/critical (rather than constructive/supportive) or an absence of workplace supervision are strong predictors of healthcare workers intentions to leave their positions [2]. Supervision that consisted mainly of negative feedback was viewed to be almost as de-motivating as no supervision. Those who indicated they only receive negative supervision were more likely to state intention to leave their jobs and negative supervision was also linked with lower levels of job satisfaction. However, health managers commonly neglect supervision, and many supervisors lack the knowledge, skills and tools for effective supervision [3].

Despite its’ critical role, supervision and in particular supportive supervision is not well understood in the context of healthcare [4, 5]. In many resource-constrained countries, the traditional visiting inspector model of supervision is common, yet there is broad consensus that this is not effective [6, 7]. District Health Management Teams (DHMT) schedule regular visits to each facility, but the evidence shows that these frequently do not happen as planned. Challenges to effective supervision include conflicting responsibilities and demands on supervisors’ time, inadequate finances and transport and accessibility problems [5]. This can result in remote facilities receiving only one visit a year. Given that external supervisory visits are sometimes their only link to more experienced health professionals, this can leave staff in rural facilities feeling abandoned and isolated.

It is clear that the existing supervision paradigm in resource-constrained countries is inadequate. Models of more supportive supervision processes stress activities that are cognisant of the underlying drivers of health worker motivation, job satisfaction and performance. A systematic approach to developing strong Human Resource Management (HRM) functioning within the health system, with improved mechanisms for management support where healthcare workers receive constructive supervision and feedback aimed at improving performance, is needed to increase retention and improve quality of care.

A focus on supportive supervision engenders a mindset where teams of health workers identify their own challenges and achieve results with support from their supervisors. Direct, on site supervision is the most effective for improving staff performance, but supervisors need to have the skills to undertake this role. Previous research [5] provides evidence that many managers and facility in-charges have not received training in HRM or supervision. In Mozambique, the personnel charged with managing health care workers currently do not have the training for effective management of the workforce [3]. Without these skills, it is difficult for them to support staff or to find the root causes of poor performance.

In this context, the aim of the Support, Train and Empower Managers (STEM) study was to develop and test, through a cluster controlled trial, an intervention to improve retention and motivation and facilitate staff to be more engaged in and ultimately more satisfied in their jobs. By improving the supervision skills of staff having a supervisory role within facilities, STEM expected to achieve greater satisfaction and improved performance of workers.

## Methods

**Fig. 1:**
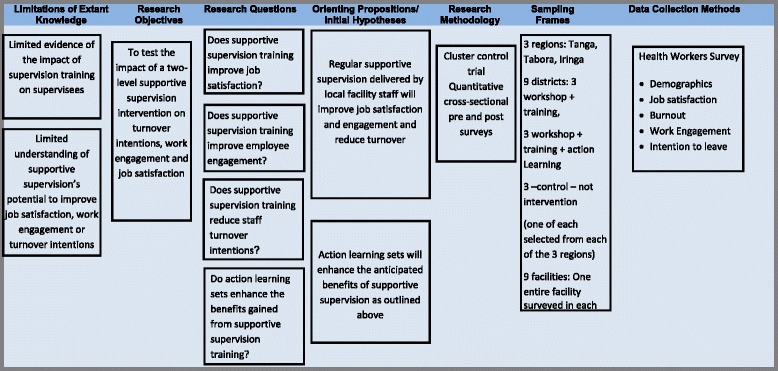
The research framework

The overall objective of the STEM project (Support, Train and Empower Managers) was to strengthen the human resource management (HRM) function at district and health facility level, by increasing the capacity of managers to support and supervise their staff. The project was implemented in Mozambique by Community Health Department of the Faculty of Medicine of University Eduardo Mondlane (UEM) and the National Health Institute (NHI), in collaboration with the Centre for Global Health, Trinity College Dublin. The research framework (Fig. 1) sets out the two orienting propositions on which this study is based:

- Regular supportive supervision delivered by local facility staff will improve job satisfaction and engagement and reduce turnover.
- Action learning sets will enhance the anticipated benefits of supportive supervision as outlined above.

Figure 1 also lists the key research questions and data collection methods.

### Study design

STEM used a cluster controlled design to evaluate the impact of the following steps:

(A)  Workshops with district health management teams and facility managers on human resource management
(B)  Intensive training in supervisory and support skills for managers directly engaged in supervision
(C)  Action learning sets for staff engaged in supervision at district and facility level

The design included three groups, with two intervention groups and one control group as follows:

- Group 1: Intervention with steps A and B = A + B
- Group 2: Intervention with steps (A), (B), and (C) = A + B + C
- Group 3: No intervention = control

### Sampling

Niassa Province was selected as the site for this research. As a remote, sparsely populated province, it often experiences considerable difficulties attracting health workers and the geographical spread of the health facilities creates additional challenges for effective supervision of the health workers that are in post. Within the province, three districts were selected to take part in the intervention, namely Mecanhelas, Mandimba and Cuamba. The selection of the districts was discussed with the Niassa Province Health Directorate and the following influenced the final decision:

- Nearly 50% of the Niassa Province population reside in the three selected Districts
- Work with facilities where few or no other non-governmental or governmental organisations were currently providing supervision training
- Include facilities that have greater numbers of healthcare staff to ensure a sufficient sample size
- Include geographically dispersed health facilities which was deemed important to reduce the risk of cross-contamination between groups located in neighbouring districts

In the two intervention districts, seven health facilities were selected to receive the intervention. In Cuamba, which served as the control district, four facilities were selected for collection of baseline and end-line data. Table 1 provides details on the particular districts assigned to each of the study groups. All health facilities in each of the selected districts were assigned to the same group or *Intervention—component A*.

**Table 1:** Summary of participants, methods and instruments/themes

Participant population	Sample size	Method	Instruments/themes
Health workers	92 (baseline) 49 (post-intervention)	Health Worker survey	Demographics Experience of supervision Supervisors Self-Assessment Inventory (adapted) Warr-Cook Job satisfaction scale Intention to leave (3-item previously validated scale) Maslach’s Burnout Inventory (Emotional Exhaustion scale) Utrecht Work-Engagement Scale
Health workers	16	Interviews	Supportive supervision; problem-solving; impacts on performance, confidence, motivation and job satisfaction; changes in the work environment

During the workshops in the districts of Mecanhelas and Mandimba, the following topics were covered:

- Introduction to STEM project
- Action Learning Sets
- The role of HR management in health systems
- Managing and working with people
- Introduction to supervision
- Supporting supervisors

A total of 25 participants from the two intervention districts participated in the workshops, 14 from Mandimba and 11 from Mecanhelas district. These included the directors of health, mother and child health programme district in-charges, district in-charge of nursing, heads of Expanded Programme of Immunisation (EPI) and HIV/AIDS programmes, supervisors of laboratories and pharmacies, district human resources managers, district in-charge of community health and head of Malaria programme, chief nurses of maternity wards and in-charges of health centres. The workshops were facilitated by members of the research team from Eduardo Mondlane University and the National Health Institute Mozambique with expertise in training healthcare staff in human resources management.

### Intervention—component B

The training (intensive training in supervisory and support skills for managers directly engaged in supervision) was carried out in April 2013. The training aimed to strengthen the capacity of the participants to conduct internal supportive supervision at their health facilities to improve provision of health care services, build the knowledge, skills and attitude of the participants and so enable them to improve their supportive supervision practices and consequently make positive impacts on health worker and facility performance. Different training methods were used to achieve the goal of the programme which includes lectures, simulations, case studies, demonstrations, assignments, plenary presentations and discussions. The delivery of the training was flexible and aimed at enabling participants to familiarise with all aspects of the key issues. A total of 36 health workers took part in the training, 19 from Mandimba and 17 from Mecanhelas. Participants to the training were District Medical Officer (DMO), Reproductive and Child Health (RCH) Coordinator, heads of RCH, health facilities in-charge, nurses in-charge of maternity wards, and programmes/sectors supervisors, namely pharmacy, laboratory, EPI, community health, tuberculosis and human resources. It was observed that most of the supervisors based at the main health facility were carrying out local and district roles because of the scarcity of health workers in the overall system.

### Intervention—component C

In group 2 intervention districts (i.e. workshop + training + ALS), supervisors who attended the intensive training are also taking part in Action Learning Sets (ALS). Action learning sets are a proven methodology for sustaining change initiatives in organisations. The purpose of these facilitated learning sets (who meet on a monthly basis) is to support collaborative learning and problem solving, building on the learning from the STEM intensive training and allowing participants to discuss challenges and successes in implementing their new supervisory skills. In effect, they form a peer group network within the district that can provide mutual support and back up, as well as sharing best practice and lessons learned. Nineteen supervisors from Mandimba district, who participated in the intensive training, participated in the STEM ALS.

### Data collection

The baseline data collection took place in July 2012 and the end-line data collection took place in April/May 2014, with the intervention components (workshops + training+ ALS) running from July 2012 to April 2014. All health workers who were present in the facility on the days of data collection were invited to partake in the study.

#### Measures

The STEM intervention was aimed at having a positive impact on health workers’ experience of supervision and on their levels of job satisfaction and engagement as a result of positive changes in the behaviour of their supervisors. A pre- and post-intervention health worker survey was designed to assess health workers’ perceptions of their jobs and their experiences of supervision. This paper focuses on the impact of the intervention on job satisfaction, burnout, work engagement and intention to leave.

#### Health worker survey

This was a self-administered survey for supervisees. The target group for this instrument was health workers in all sampled facilities in the project districts where the intervention had been conducted with the facility-in-charges and supervisors.

Section A: contained demographics, medical qualifications, current job position and length of service in the current facility

Section B: contained information on the supervisee’s experience of supervision (the results of this will be reported elsewhere

Section C: A corresponding instrument to the Supervisors Self-Assessment Inventory was used to assess health workers’ perceptions of their supervisor’s performance. (The results will be reported in a forthcoming publication.)

Section D: measured job satisfaction using the Warr-Cook-Wall Job Satisfaction Scale [8]. It has become one of the most commonly used measures of job satisfaction and has been employed across a range of occupational groups, both in the original 16-item form (used in this study) or in adapted, shortened versions. Participant responses are usually captured on a 7-point Likert scale and scored from 1 to 7, with 1 representing the most negative response. Our previous research and piloting of this instrument demonstrated that the 7-point Likert scale format did not work well with this population as participants tended to opt for the mid-points and not use the full extent of the scale. For the purpose of this study, we therefore adapted it to a 3-point format—I am unhappy, I am neither happy nor unhappy and I am happy and adjusted the scoring accordingly.

Section E: measured participants’ intention to leave their current position, using three items:

- I have never thought about leaving this health facility
- I have seriously thought about leaving this health facility
- I am actively seeking other employment.

Responses are captured on a 5-point Likert scale, ranging from strongly disagree to strongly agree. These items proved a robust and reliable measure in previous studies with similar populations [1, 9].

Section F: measured levels of burnout using the 9-item Emotional Exhaustion subscale of Maslach’s Burnout Inventory (MBI) [10]. Responses are given on a 7-point scale (never, a few times per year, once a month or less, a few times per month, once a week, a few times per week and every day). High scores on the emotional exhaustion scale represent greater burnout. The MBI is the gold-standard questionnaire in this area and has been cited in over 1000 studies. It is increasingly being used with health workers in Africa (e.g. Malawi, Nigeria, Ghana [11]. Two of the three subscales were excluded (based on poor performance of these scales in Tanzania in a previous study) and only the Emotional Exhaustion sub-scale was administered.

Section G: uses the 17-item Utrecht Work Engagement Scale (UWES) to measure work engagement, [12] which is described as ‘…a positive, fulfilling, work-related state of mind that is characterized by vigour, dedication, and absorption.’ [13]. The UWES subscales measure these items.

- Vigour (six items—G1, 4, 8, 12, 15, 17) refers to high levels of mental energy and resiliency whilst working and personal investment at work.
- Dedication (five items—G2, 5, 7, 10, 13) describes feelings of pride, meaningfulness, challenge and enthusiasm about one’s work.
- Absorption (six items—G3, 6, 9, 11, 14, 16) refers to being fully immersed in one’s work and losing all sense of time whilst working.

Work engagement is conceptually linked to burnout, with studies demonstrating a negative correlation between engagement and burnout, particularly vigour (UWES) and exhaustion (MBI) [14]. It is theorised to have high pleasure and activation levels, whereas burnout is characterised by low pleasure and activation (e.g. [15]). However, low burnout does not necessarily mean high work engagement or vice versa. The UWES’s three-factor model (i.e. vigour, dedication and absorption) has demonstrated acceptable internal consistencies with a South African police population, (Storm et al. [16]) but has not been well used in sub-Saharan African contexts.

#### Health worker interview

The STEM end-line data collection also included interviews with health workers whose supervisors had been part of the STEM training programme. These aimed to explore whether this training had had any impact on staff and to identify any changes they had experienced in the last 12 months. Areas of interest were changes in how facility and ward in-charges conduct internal supportive supervision; problem-solving; impacts on performance, confidence, motivation and job satisfaction; and changes in the work environment (feedback, standards and goal setting, access to training/skills support, teamwork, participation and voice, ethics and accountability). Staff were invited to partake if they had worked in the current facility for at least 12 months and had not taken part in the STEM training themselves. Seven interviews were conducted in each of the A + B and A + B + C districts. Two further interviews were carried out in the control district, providing a total of 16 interviews.

## Results

### Demographics

There were a total of 92 health workers sampled at the baseline and 49 at the end-line from health facilities in three districts within Niassa province—Cuamba, Mecanhelas and Mandimba. Table 2 shows the number of health workers (in brackets) sampled from each health facility and each district at the baseline and end-line. The distribution of health workers across intervention districts differed a little at the baseline and end-line. At the baseline, over half of sampled health workers were from the control district Cuamba (52.2%), with just under one quarter (23.9%) from Mandimba (intervention A + B + C district). At the end-line, health workers were more evenly distributed across Cuamba (36.7%) and Mandimba (40.8%). In both samples, health workers from the intervention district A + B made up a little less than one quarter of the sample, with only 11 health workers from this district in the end-line sample.

**Table 2:** Distribution of health workers across regions, districts and health facilities

District	Stage of data collection	
	Baseline	End-line
^a^Cuamba	48 (52.2%)	18 (36.7%)
^b^Mandimba	22 (23.9%)	20 (40.8%)
^c^Mecanhelas	22 (23.9%)	11 (22.4%)
Total	92 (100%)	49 (100%)

^a^Group 3—district is acting as a control in the study

^2^Group 2—interventions A, B and C are being implemented in this district

^c^Group 1—interventions A and B are being implemented in this district

**Fig. 2:**
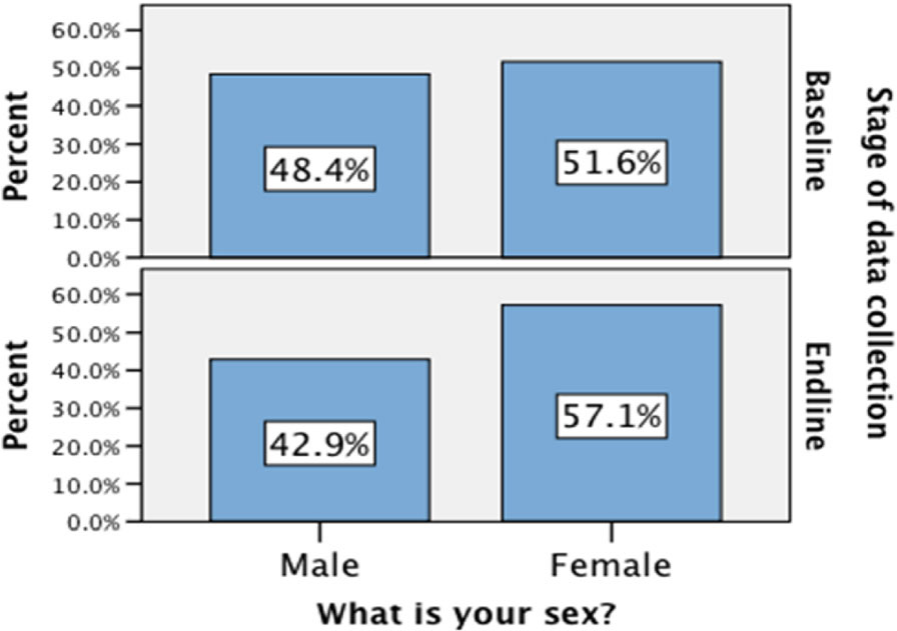
The distribution of gender at the baseline and end-line

Age profiles were broadly similar at the baseline and end-line, although the baseline sample was a little older on average with a mean age of 34.4 years (SD = 8.3) than the end-line sample with a mean age of 32.3 years (SD = 8.0). The distribution of cadre levels was quite similar at the baseline and end-line. There was a slightly higher percentage of mid-level cadres at the end-line (65.3%) than at the baseline (54.2%) and a slightly lower percentage of elementary and basic level cadres at the end-line. The distribution of gender at the baseline and end-line was broadly similar (see Fig. 2).

### Data analysis

Mann-Whitney *U* test is the alternative test to the independent sample *t* test. It is a non-parametric test of the null hypothesis that it is equally likely that a randomly selected value from one sample will be less than or greater than a randomly selected value from a second sample used to compare two population means that come from the same population. It is also used to test whether two population means are equal or not. Unlike the *t* test, it does not require the assumption of normal distributions. In this study, it was used to compare the pre- and post-intervention data (on non-matched samples from the same population of health workers) and to compare the post-intervention data for the intervention groups with the control group. These tests were conducted for each construct but detailed outputs are provided only where results reached acceptable levels of significance.

### Job satisfaction

**Fig. 3:**
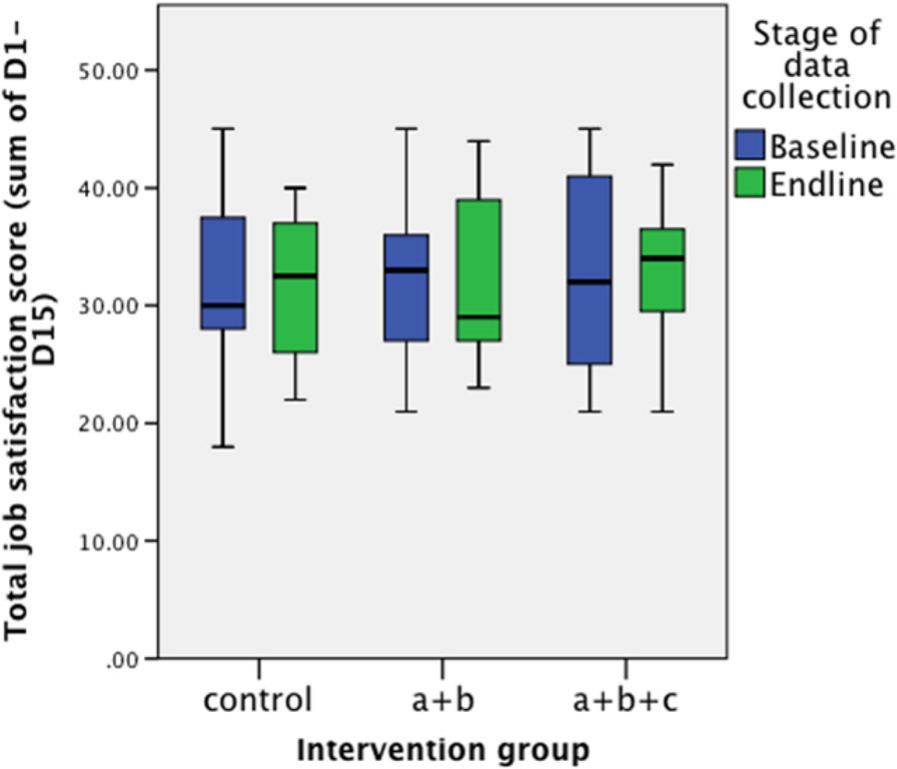
The spread of scores across the different trial arms pre- and post-intervention

The Mann-Whitney *U* tests on the changes in total job satisfaction scores found no statistical significant differences between the pre- and post-intervention measures for any of the cohorts in the sample (Table 3). STEM recorded some changes (that did not reach significance level) in health worker job satisfaction; however, the direction of these changes varied. Figure 3 shows the spread of scores across the different trial arms pre- and post-intervention. Health workers were asked about satisfaction with their job. There are some differences between the baseline and end-line samples. However, the direction of these differences was mixed. For example, 23.3% of the baseline sample responded that they were not happy with their jobs overall, whilst at the end-line, this percentage was almost half that, at 12.9%. In contrast, whilst 46.5% of the baseline health worker sample responded that they were happy with ‘how interesting [their] job is’, this percentage was much lower at the end-line (25.8%).

**Table 3:** Differences is mean job satisfaction scores between the baseline and end-line

Intervention group	Difference in sample means (end-line–baseline)	Standard error of the difference in sample means	Difference in sample medians (end-line–baseline)	Mann-Whitney *U* test
Control	−0.3	1.6	2.5	*U* = 409 *Z* = −0.33 *p* value = 0.74
Intervention A + B	−0.3	2.6	−4	*U* = 114 *Z* = −0.27 *p* value = 0.78 9
Intervention A + B + C	0.4	2.2	2	*U* = 200 *Z* = −0.26 *p* value = 0.79 4

### Intention to leave

**Fig. 4:**
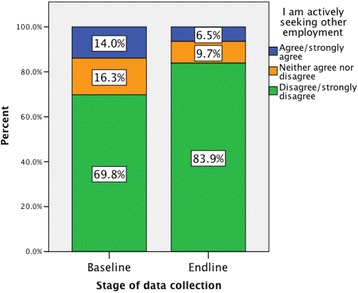
The number of health workers actively seeking other employment reduced after the intervention

The number of health workers actively seeking other employment reduced after the intervention (see Fig. 4). At the end-line, a considerably higher percentage of health workers (83.9%) disagreed or strongly disagreed that they were actively seeking other employment, than at the baseline (69.8%). However, Mann-Whitney *U* tests found no statistical significant differences between the pre- and post-intervention measures for any of the cohorts in the Mozambique sample (Table 4).

**Table 4:** Differences in mean scores on intention to leave at the baseline and end-line

Item		Intervention group	Difference in sample means (end-line–baseline)	Standard error of the difference in sample means	Difference in sample medians (end-line–baseline)	Mann-Whitney *U* test
E1	I have never thought about leaving this health facility	Control	0.48	0.38	1	*U* = 353.5, *Z* = −1.16, *p* value = 0.245
		A + B	0	0.53	0	*U* = 117, *Z* = −0.16, *p* value = 0.87 3
		A + B + C	−0.55	0.41	−1	*U* = 157, *Z* = −1.44, *p* value = 0.14 9
E2	I have seriously thought about leaving this health facility	Control	−0.07	0.35	−0.5	*U* = 416, *Z* = −0.24, *p* value = 0.80 7
		A + B	−0.14	0.46	−0.5	*U* = 111, *Z* = −0.42, *p* value = 0.67 8
		A + B + C	−0.04	0.43	1	*U* = 204, *Z* = −0.17, *p* value = 0.86 7
E3	I am actively seeking other employment	Control	0.21	0.35	0.5	*U* = 403, *Z* = −0.45, *p* value = 0.65
		A + B	−0.09	0.48	0	*U* = 111, *Z* = −0.4, *p* value = 0.68 9
		A + B + C	−0.36	0.3	0	*U* = 184, *Z* = −0.81, *p* value = 0.42

### Burnout

Health workers in the end-line sample seemed to score lower on individual items than in the baseline. When asked about the frequency with which they felt ‘emotionally drained from [their] work’, 51.3% of the baseline sample responded that they felt this at least a few times per month, compared with 45.1% of the end-line sample. When asked about how often they ‘feel used up at the end of the workday’, 30.9% of the baseline health workers responded that they felt this at least a few times per month, compared with the lower percentage (23.3%) of health workers surveyed at the end-line.

**Fig. 5:**
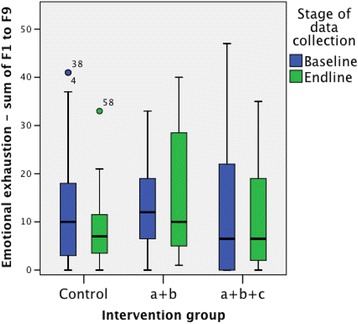
Total scores on emotional exhaustion

Total scores on emotional exhaustion were positively skewed at both the baseline and end-line for all intervention groups, with many health workers scoring low on emotional exhaustion, and a large spread of values above the median (Fig. 5). However, there are little or no differences evident in the distributions of the baseline and end-line scores.

Mann-Whitney *U* tests did not show statistically significant differences in the distribution of emotional exhaustion scores between the baseline and end-line samples, for any of the intervention groups.

Emotional exhaustion scores were also categorised as low (0–16), moderate (17–26) or high (≥27) as recommended in the MBI manual [10]. Table 5 shows the percentages of health workers falling into each category. For intervention A + B + C, the percentage of health workers with a high level of emotional exhaustion was lower at the end-line (5.6%) than at the baseline (22.2%). A similar result was seen in the control arm, whilst for intervention A + B health workers, there was a greater percentage at the end-line with a high level of emotional exhaustion than at the baseline.

**Table 5:** Categories of emotional exhaustion

Intervention	Level of emotional exhaustion	Baseline	End-line
		*n* (%)	*n* (%)
Control	Low	31 (68.9%)	13 (81.2%)
	Moderate	7 (15.6%)	2 (12.5%)
	High	7 (15.6%)	1 (6.2%)
A + B	Low	13 (68.4%)	6 (54.5%)
	Moderate	3 (15.8%)	2 (18.2%)
	High	3 (15.8%)	3 (27.3%)
A + B + C	Low	11 (61.1%)	13 (72.2%)
	Moderate	3 (16.7%)	4 (22.2%)
	High	4 (22.2%)	1 (5.6%)
All	Low	55 (67.1%)	32 (71.1%)
	Moderate	13 (15.9%)	8 (17.8%)
	High	14 (17.1%)	5 (11.1%)

### Work engagement

For the UWES, responses on some items differed for the baseline and end-line health worker samples. For example, 15% of the baseline health workers responded that their job never inspires them, whilst none of the end-line health workers felt this. Health workers were asked about the frequency with which they felt that ‘Time flies when [they are] working’—at the baseline, 84.6% responded that they felt this at least a few times per month, whilst at the end-line, 93.5% of health workers provided the same response. Overall scores on vigour, dedication and absorption were slightly higher at the end-line, but this was equally the case in the control and the intervention groups. The results of Mann-Whitney *U* tests for differences in distribution of total scores showed no statistically significant differences in distribution of total scores on vigour, dedication and absorption between the baseline and end-line health worker samples in Mozambique for any of the intervention groups.

### Health workers interview

Analysis of the qualitative material from the health worker interviews identified perceptions of considerable improvement in supervisors’ performance. The key themes emerging from the data are presented in the following section.

### Performance

Performance was a key theme in participants’ responses. Health workers could see the improvements to their own performance and credited it to supervision. ‘When he supervises me I like it very much because there is no service that a person can do without supervision. Here we work together and also, when there is supervision it motivates the person to change.’ (1–14)^1^

Another interviewee describes the improvement as follows:

‘I won it {a prize} because when I started working here the rate of institutional deliveries was 50% below the expected, but after the supervision we improved it… our weekly supervision sessions helped with it. We also met {stakeholders} … and all of them helped us to reverse the scenario.’ (2–24)

### Motivation

In addition, supervision was perceived by the health workers as having a positive impact on motivation. ‘It contributes…she [the supervisor] made us realise that when we are working in the end the benefits are for ourselves as well as the population we serve’. She says: ‘Imagine if you lose a job today, the number of people who will miss you because you were contributing greatly to their health and well-being.’ (1–16)

A health worker in an A + B facility thought supervision helped because ‘It’s good to have someone there to appreciate your work, because when that happens it makes you want to improve’. (2–13)

### Participation and voice

One of the more unexpected changes to the work environment, and one which participants directly attributed to the supervision intervention, was the increase in participation and voice amongst health workers in intervention facilities. When asked about changes to their work environment, health workers described a more open and participative environment, saying that there was now space for them to express their ideas and be heard. ‘…we contribute with ideas….we are listened to. He introduces what we have to discuss and then each says what he/she feels about the work…he also asks how we feel about him, the way he treats us, and we all give our opinions.’ (1–15) Another said, ‘Everybody talks freely, without fear…every idea is valued, if it’s a good idea they use it.’ (2–21)

## Discussion

The end-line sample of health workers was just a little more than half of the baseline, one reason for this poorer response may be the fact that many staff were engaged in a bed net distribution activity when the team was collecting the final data and therefore were not in the facilities to respond to the surveys. In addition, this is a region where retention is a problem and there are high absenteeism rates. Despite efforts to increase the sample size through return visits, the reality was that staff were not present in the numbers we required. In addition, at the baseline, more than half of the total sample were in the control district, thus the baseline numbers for each of the intervention groups was smaller than desirable.

The age and gender distribution across the time lines was broadly similar, and at both times, the mid-level cadres made up the overwhelming majority of the sample. The mean length of time health workers had worked in their facilities was higher at the baseline than the end-line. A lower percentage of health workers in the end-line sample had worked in their health facilities for 5 years or less than in the baseline sample. There were also substantially fewer health workers in the A + B + C group who had worked 5 years or more in the end-line sample as compared to the baseline sample. The imbalance between more and less experienced workers may create an imbalance between demand for and supply of supervision with fewer experienced workers available to offer supervision and support to the large majority of health workers who have worked in their facilities for 5 years or less.

In general, there is a relatively high turnover of staff without replacement; as 2 years post placement, health workers are entitled to proceed with further studies and/or move from remote areas to urban areas with better educational and economic opportunities.

Health workers job satisfaction scores show a mixed picture, but there are no statistically significant differences pre- and post-intervention. McAuliffe et al. [2], in a three-country study that included Mozambique, found robust evidence indicating that a formal supervision process predicted high levels of job satisfaction. The failure to reach significance in this study is most likely attributable to the small sample sizes and inability to match pre- and post-intervention samples. The McAuliffe et al. study was a cross-sectional study of more than 2000 health workers and therefore provides more robust evidence for the link between job satisfaction and supervision. This study had the more ambitious goal of demonstrating the impact of a supervision intervention in a resource-depleted and sparsely populated setting, making a sample of comparable power more difficult to attain.

Similarly, for health workers’ self-reports on ‘intention to leave’, the results are very mixed. At the end-line, health workers in intervention district A + B + C had, on average, slightly lower levels of agreement both with ‘I have never thought about leaving this health facility’, and with ‘I am actively seeking other employment’. However, statistical tests did not show significant differences in the distribution of responses on intention to leave between the baseline and end-line health worker samples, for any intervention group. Tracking attrition rates in these facilities over an extended time period would probably give a more reliable picture on whether the intervention has impacted significantly on retention. Indeed, Rouleau et al [17] in a study of 226 Senegalese midwives report that despite nearly two thirds (58.9%) of midwives reporting the intention to quit within a year, only 9% annual turnover was found in the study. Departures were largely voluntary (92%) and entirely domestic.

Another outcome measure compared before and after the intervention was emotional exhaustion and although there were slight improvements in scores, these differences were not significantly better at the end-line for any of the intervention groups or the control group. It may be that emotional exhaustion is influenced more by other factors in the environment such as workload and resource availability and that improved supervision alone is not capable of impacting on this. As the setting for this study is one of the most remote regions of Mozambique with a very low density of health workers, it is not surprising that emotional exhaustion was positively skewed. In Rouleau’s Senegalese study [17], a very large proportion of the midwives were found to have levels of emotional exhaustion classified as ‘high’ (80%) and as ‘average–high’ (94%) [17]. In East Africa, burnout amongst healthcare staff, although a problem, appears to affect fewer staff. Thorsen et al. [11] in a study of enrolled nurses in Malawi found that 33% of a sample of 101 enrolled nurses scored >27 on Emotional Exhaustion scale placing them in the high burnout category. McAuliffe et al. [9] in a study of 153 health workers of mixed cadres in Malawi, 31% of the sample were in the high emotional exhaustion category. In contrast, only 17.1 and 11.1% of the baseline and end-line health workers, respectively, sampled in this study were categorised as having high levels of emotional exhaustion.

The Utrecht Work Engagement Scale (UWES-17) was used to measure the extent to which staff were engaged in their work. Three subscales measure vigour, dedication and absorption as aspects of work engagement. Responses on some items differed for the baseline and end-line health worker samples. For example, 15% of the baseline health workers responded that their job never inspires them, whilst none of the end-line health workers felt this. Health workers were asked about the frequency with which they felt that ‘Time flies when [they are] working’—and scores on this also increased from the baseline to end-line. However, none of these differences reached statistically significant levels. Previous studies in high-income settings have consistently shown that job resources and personal resources facilitate work engagement [18, 19]. Job resources are assumed to play an intrinsic motivational role because they fulfil basic human needs, such as the needs for autonomy, relatedness and competence [20]. Several studies have shown a positive relationship between job resources and work engagement (for a meta-analysis, see Halbesleben [21]). The limited resources in many of Mozambique’s health facilities may be one factor that explains the failure of the STEM intervention to show positive effects on work engagement, the absolute scarcity of human resources in the Niassa region outweighing any potential positive impact of improvements in supportive supervision. In any case, the results need to be interpreted with caution, given that the UWES has not been validated in any comparable population and the sample size in this study prohibited validity and reliability testing with this population.

The qualitative data showed positive effects of the intervention on performance and motivation of health workers. One of the more unexpected changes to the work environment, which participants directly attributed to the supervision intervention, was the increase in participation and voice amongst health workers in intervention facilities. When asked about changes to their work environment, health workers described a more open and inclusive environment, saying that there was now space for them to express their ideas and be heard.

### Limitations

The target site, which may not in hindsight have been the most suitable, Niassa, is a rural, sparsely populated, geographically spread region that struggles to attract sufficient health workers to meet population needs. This made it challenging to obtain adequate sample sizes, exacerbated by a health promotion activity in the region. The end-line sample of health workers was just a little more than half of the baseline, one reason for this poorer response may be the fact that many staff were engaged in a bed net distribution activity when the team was collecting the final data and therefore were not in the facilities to respond to the surveys. However, the policy of allowing staff to move after 2 years of service in a remote location, regardless of whether they are replaced, could also account for the smaller numbers at the end-line. In addition, at the baseline, more than half of the total sample were in the control district; thus, the baseline numbers for each of the intervention groups were smaller than desirable. Although every attempt was made to sample adequately, this was hampered by the fact that the assigned numbers of staff per facility were not present in the facilities and we had no way of knowing this would be the case. Despite considerable effort to increase the sample, and placing a member of the research team in the province for the duration of the study, the staff were just not present at the facilities. A further limitation was the impossibility of obtaining an adequate size of matched pre- and post-sample (because of the high levels of transfer and voluntary movement of staff), thus rendering the comparative analysis less robust.

The limited size and heterogeneous nature of the sample would have rendered a re-validation of the instruments meaningless, and therefore, for instruments such as the UWES (that have not been validated in a comparable setting), the results must be interpreted with caution. Although the UWES is a widely accepted and validated instrument for the measurement of work engagement, the fact that it has not to our knowledge been validated in low-resource healthcare settings means it is difficult to interpret the study results.

## Conclusions

This paper reports on the impact of a supportive supervision intervention on health worker job satisfaction, burnout, work engagement and intention to leave. The quantitative measurements of job satisfaction, emotional exhaustion and work engagement showed no statistically significant differences between the end-line and baseline. This cannot be interpreted as meaning that the intervention was not effective as the response rates were too low and sample sizes too small to conduct adequate analysis for these metrics. However, the qualitative data collected from health workers post the intervention in the intervention districts showed many positive impacts on health workers not captured by the quantitative survey. Health workers perceived an improvement in their performance and attributed this to the supportive supervision they had received from their supervisors following the intervention. Reports of increased motivation were also common. An unexpected, yet important consequence of the intervention, which participants directly attributed to the supervision intervention, was the increase in participation and voice amongst health workers in intervention facilities. The small sample sizes make it difficult to generalise the findings. Nonetheless, the study lends support to the importance of strengthening human resource management and particularly supportive supervision in motivating and retaining health workers in Mozambique.

## Abbreviations

ALS: Action Learning Sets
DHMT: District Health Management Teams
DMO: District Medical Officer
EPI: Expanded Programme of Immunisation
HIV/AIDS: Human Acquired Immunodeficiency syndrome
HRM: Human Resource Management
MBI: Maslach’s Burnout Inventory
RCH: Reproductive and Child Health
SD: Standard deviation
STEM: Support, Train and Empower Managers
UWES: Utrecht Work Engagement Scale

## Footnotes

1 1 = Mandimba, 2 = Mecanhelas, double digit number = facility identifier. All quotes from intervention districts only.
